# Editorial: Perspectives for the Next Generation of Virus Research: Spearheading the Use of Innovative Technologies and Methodologies

**DOI:** 10.3389/fmicb.2017.00758

**Published:** 2017-05-11

**Authors:** Takatoki Yamamoto, Ayae Honda, Toshinori Sato, Akihide Ryo

**Affiliations:** ^1^Department of Mechanical Engineering, School of Engineering, Tokyo Institute of TechnologyTokyo, Japan; ^2^Department of Frontier Bioscience, Hosei UniversityTokyo, Japan; ^3^Department of Biosciences and Informatics, Faculty of Science and Technology, Keio UniversityYokohama, Japan; ^4^Department of Microbiology, Yokohama City University School of MedicineYokohama, Japan

**Keywords:** technology, nano-sensor, optical tweezer, proteomics, glycomics, bio-informatics, peptide, DNA editing technology

Science and technology are fundamental to overcome future challenges and threats to humankind. For instance, infectious diseases have emerged and re-emerged as a major global hazard in the early twenty-first century. This threat has become accepted as one of the most important issues to be tackled by science. Technological innovations have led to a better understanding of ongoing scientific challenges such as those posed by viruses. Our research topic aims to advance the field of virology by promoting cutting-edge technologies and technology convergence that have not been commonly used before. The technologies that could be cataloged in this novel collection include nanobiosensors, nanopore/nanochannel detectors, optical tweezers, functional proteomics, functional glycomics, trans-omics informatics, peptide-based drug discovery, next-generation sequencing and DNA editing technology.

This Research Topic contains 3 review articles, 1 mini-review article, 1 data report and 15 original research articles.

Miyake et al. at first research article identifies the host factor pyruvate kinase M2 that interacts with the PA subunit of RNA-dependent RNA polymerase (RdRp), which is vital for transcription and replication of influenza virus. This article integuments molecular mechanism of host-virus interaction and offers the exciting insight into the development of new therapeutic approaches. The review article by Yang and Yamamoto provides the overview of the sophisticated nanopore based electrical sensing techniques for quantitative detection of virus particles. Here the authors show that nanostructure-based electrical sensors may use for real-time, sensitive detection of new and unknown virus particles in the absence of biochemical information. In the third issue the authors (Liu et al.) depict that extracellular pH reduces near cell membrane followed by influenza virus infection. In this study they develop a sensor based on Rhodamine B and FITC fluorescence for measurement of pH changes. Cho et al. uses the fluorescent polymeric nanoprobes for observing the levels of sialic acid receptors expressed on cell membrane to examine the progression of influenza viral infection. Human Immunodeficiency Virus (HIV) infection causes integration of proviral DNA into the chromosomes that potentially leads to reemergence of virus. In the fifth research article Kishida et al. manifests that this proviral DNA can be destructed by genome editing technology. In the sixth subject the authors explore a novel fact for entry of HSV-1 into the Chinese hamster ovary (CHO-K1) cell using lipofectamine transfection reagent (Burnham et al.). Glycoprotein-D receptor is mainly responsible for HSV-1 entry into the cell but here they delineate that HSV-1 is entered into CHO-K1 cell independent of gD receptor.

Jo et al. publishes a data report for complete genome sequencing of Dengue Virus (DENV) 3 from the *Pythium insidiosum* transcriptomes. In this data report the authors use several modern and new techniques such as NGS, MEGA BLAST, FASTA which give incredible acquaintance to the readers for future virus research. The research article by Kudoh et al. develops a new proteomic approach to study virus host interaction. They show how host regulatory factor HSPB8 negatively affects the replication of HIV-2 that may be decisive for preventing placental infection of HIV-2 during pregnancy. In the ninth case Yamashita et al. designs a database Dengue Genographic Viewer (DGV) which is freely available at online. DGV covers the geographical distribution of DENV genotype and serotype in a user specified time span. DGV may aid in planning to scientists for the control of DENV infections. Yuan and Gao identifies several phage structural proteins by structural proteome analysis. The review article by Mirvish and Shuda aims at deciphering the discovery of the human tumor viruses using Digital Transcriptome Subtraction (DTS) than other conventional microscopic observation. The focus is how DTS developed and used for the discovery of merkel cell polyomavirus (MCV). In the 12th research article Kamba et al. represents the methodology for the real time NMR spectroscopy and the ease of its usage and legibility to systems having different temporal and spatial resolution. In the 13th case as original research article Yamaoka et al. develop a novel, rapid and reliable antigen detection assay for MERS coronavirus using the monoclonal antibodies. They produce the monoclonal antibodies by wheat germ cell free system using NP antigen of MERS-CoV. This study serves the necessary cue that the wheat germ cell-free system is useful for the development of diagnostic mAbs against new emerging pathogens. Cao et al. at 14th research article establishes a method to diagnose WNV using isothermal amplification combined with a vertical flow visualization strip. This assay is rapid, simple and suitable for clinical application in the field. In the next article Imamura et al. focuses an approach to detect viruses with nanomechanical sensors. The research article by Matsubara et al. depicts a penta peptide of lipid membrane that binds to hemagglutinin (HA) during influenza virus infection by mimicking sialic acid. Chang et al. review the significance of filopodia in viral diseases. Here the authors overview the key determinants of filopodial induction and their influence on cell topography including receptor expression for viral entry. In the 18th review article Zhang et al. provides an overview of virus derived small interfering RNAs (vsiRNAs) pathways focusing the advances in vsiRNAs biogenesis and function in the field of plant virus research. In the 19th research article Matsunaga et al. develops a novel *in vitro* method, the cell-free drug susceptibility assay (CFDSA), for monitoring phenotypic information regarding the drug resistance of HIV-1 protease (PR). The CFDSA utilizes a wheat germ cell-free protein production system to synthesize enzymatically active HIV-1 PRs. Finally in the last issue Hatsuki et al. in the original research article proposes a virus detection method by nonlinear impedance spectroscopy under a strong electric field between two nanogap electrodes.

In summary, it is concluded that these works are well outlined to flourish the new and innovative approaches understanding the basic and clinical aspects of the technology-based virus research. We are optimistic that this collection of papers will eventually boost the knowledge of readers and serve as fundamental frameworks for future virus research.

## Author contributions

All authors listed, have made direct and intellectual contribution to the work, and approved it for publication.

## Funding

This work was supported in part by a Creation of Innovation Centers for Advanced Interdisciplinary Research Areas Program and JSPS Grants-in-Aid for Scientific Research (16H05198) to AR.

### Conflict of interest statement

The authors declare that the research was conducted in the absence of any commercial or financial relationships that could be construed as a potential conflict of interest.

